# Prevalence and Genotyping of Water- and Food-Borne Parasitic Protozoans (*Giardia duodenalis* and *Cryptosporidium* spp.) in Hospitalized Patients from Northwestern Romania

**DOI:** 10.3390/microorganisms12040762

**Published:** 2024-04-10

**Authors:** Angela Monica Ionică, Anca Ieremia, Zsuzsa Kalmár, Mihaela Lupșe, Mirela Flonta, Monica Muntean, Cristina Cismaru, Melinda Horvat, Amanda Rădulescu, Adriana Topan, Cristian Jianu, Georgiana Deak, Violeta Briciu

**Affiliations:** 1 Clinical Hospital of Infectious Diseases of Cluj-Napoca, 400348 Cluj-Napoca, Romaniamihaela.lupse@yahoo.com (M.L.); m.flonta@yahoo.com (M.F.); monica.muntean@umfcluj.ro (M.M.); cristian_jianu@yahoo.co.uk (C.J.);; 2 Department of Infectious Diseases, “Iuliu Haţieganu” University of Medicine and Pharmacy, 4000348 Cluj-Napoca, Romania; 3 Department of Microbiology, Immunology, and Epidemiology, University of Agricultural Sciences and Veterinary Medicine of Cluj-Napoca, 400372 Cluj-Napoca, Romania; 4 Department of Parasitology and Parasitic Diseases, University of Agricultural Sciences and Veterinary Medicine of Cluj-Napoca, 400372 Cluj-Napoca, Romania; georgiana.deak@usamvcluj.ro

**Keywords:** *Cryptosporidium* spp., *Giardia duodenalis*, genotyping, symptoms, Romania

## Abstract

*Giardia duodenalis* and *Cryptosporidium* spp. are important zoonotic protozoan pathogens that infect the gastro-intestinal tract of numerous vertebrates, including humans, and both parasites are responsible for water- or food-borne outbreaks of disease worldwide. Although, globally, both parasites are highly prevalent, particularly in developing countries, epidemiological data from Romania are scarce, and genotyping has rarely been performed. The aims of the present study were to investigate the occurrence and genetic diversity of *G. duodenalis* and *Cryptosporidium* spp. in patients hospitalized in Northwestern Romania in relation to clinical and paraclinical presentation and to identify the relative frequency of non-specific symptoms and potential risk factors. Between June 2022 and January 2024, 426 fecal samples were screened for gastro-intestinal parasites by rapid tests and microscopical examination, further confirmed by PCR and sequencing. *Giardia duodenalis* was detected and characterized in 12 samples (2.82%), while *Cryptosporidium parvum* was confirmed in four samples (0.94%). A majority of positive patients were symptomatic and reported nausea and vomiting with a significantly higher frequency compared to negative ones. This study provides new insights into the epidemiological status and clinical implications of gastro-intestinal parasite species and genospecies in Romania that are necessary for an in-depth understanding of the potential zoonotic transmission and improvement of patient care.

## 1. Introduction

*Giardia duodenalis* and *Cryptosporidium* spp. are important zoonotic protozoan pathogens that infect the gastrointestinal epithelium of a wide range of vertebrate hosts, including humans. The environmental stages of both parasites (*G*. *duodenalis* cysts and *Cryptosporidium* spp. oocysts) are shed in feces, being transmitted by the fecal–oral route, either via the ingestion of contaminated water or food, or by contact with contaminated surfaces and materials [1,2]. Both parasites are highly resistant to disinfectants; thus, as a consequence, they are responsible for numerous water- or food-borne outbreaks of disease worldwide [1,3]. 

*Giardia* spp. are diplomonad flagellates that belong to the family Hexamitidae. Of the nine recognized species, only the *G. duodenalis* (syn. *G. intestinalis*, *G. lamblia*) species complex causes human infection [1,4]. The *G. duodenalis* complex comprises eight established genotypes (A to H), referred to as assemblages in mammals [1], with assemblages A and B being responsible for almost 95% of human infections [5]. Infections may frequently be asymptomatic or cause mild illness, and they usually resolve without treatment. The clinical presentation mainly includes nausea, diarrhea, abdominal cramps, vomiting, and bloating. Some individuals may experience chronic infections, which have been associated with irritable bowel syndrome (IBS), food allergies, chronic fatigue syndrome, and arthritis [6].

Although the taxonomic classification of the genus *Cryptosporidium* is still unclear and requires further revision [1], currently 22 species and two genotypes have been reported to cause human infections, among which *C. hominis* and *C. parvum* were responsible for the great majority of cases [3]. The pathogenicity of *Cryptosporidium* varies with the species and the type, but host-related factors, such as age and immune status, also influence the clinical presentation [7]. The most common clinical presentation of cryptosporidiosis is profuse watery diarrhea with abdominal pain, low-grade fever, nausea, vomiting, and weight loss. It can often be asymptomatic, mild, or self-limiting, lasting approximately 5–10 days, in immunocompetent individuals [8]. However, in young and/or immunocompromised individuals, the infection can cause severe, chronic diarrhea, leading to malabsorption, with long-term negative effects on the growth and cognitive development of children [9]. 

*Giardia duodenalis* is one of the most prevalent enteric parasites globally, having a prevalence of up to 33% in developing countries and an estimated incidence of around 280 million new cases annually [1,10]. In Romania, the actual frequency of giardiasis is mostly unknown, with reported regional prevalence values ranging between 2 and 25% in symptomatic patients [11]. A screening survey performed in Northwestern Romania indicated a prevalence of 0.42% in seemingly healthy, asymptomatic patients [12]; it was the only study to address the genotyping of this parasite in the country. Globally, the prevalence of *Cryptosporidium* spp. is estimated to be 7.6%, with an average of 4.3% in developed countries and 10.4% in developing countries [13]. In Romania, studies performed between 1980 and 1996 indicated prevalence values ranging between 1.8 and 12.3% in children [14]. Between 2008 and 2021, a total of 27 human cases were officially reported for Romania by the National Authorities, indicating significant underreporting of the cryptosporidiosis [15,16,17]. Genetic data on human isolates are scarce, with a total of nine specimens characterized till date, originating from patients with diarrhea who were hospitalized in the western part of the country [18,19]. 

The aims of the present study were to investigate the occurrence and genetic diversity of *G. duodenalis* and *Cryptosporidium* spp. in patients hospitalized in Northwestern Romania in relation to clinical presentation and hematological parameters and to identify the relative frequency of non-specific symptoms and potential risk factors. 

## 2. Materials and Methods

The study was conducted in accordance with the Declaration of Helsinki and approved by the Institutional Ethics Committee of Clinical Hospital of Infectious Diseases of Cluj-Napoca through Decision 8899, from 13 May 2022.

The study took place between June 2022 and January 2024, at The Clinical Hospital of Infectious Diseases, a large Tertiary Center located in Cluj County. All non-COVID patients admitted during this period were given the opportunity to participate. Upon verbal agreement, a written informed-consent form and a questionnaire (Supplementary Materials) were provided to be filled out and signed by the participant or legal guardian in the case of minor patients. For each participant, the admission diagnosis and the results of the complete blood count (CBC) performed upon hospital admission (as per standard of care) were recorded. 

**Figure 1:**
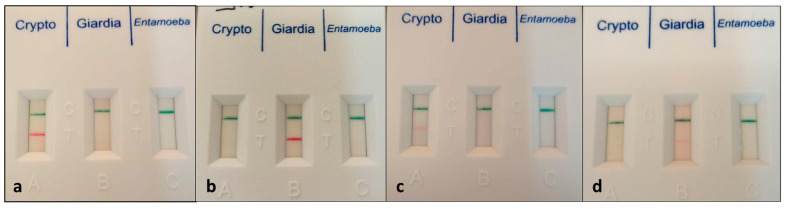
Visual assessment of test band intensity: positive (**a**,**b**) and inconclusive (**c**,**d**) samples.

For each participant, three consecutive stool samples were collected. The samples were stored at 4 °C and processed immediately after collecting the third one. Firstly, a rapid test able to identify antigens of *Cryptosporidium*, *Giardia duodenalis*, and *Entamoeba histolytica/dispar* (ONE STEP *Cryptosporidium*, *Giardia*, and *Entamoeba* COMBO CARD TEST, CerTest Biotec, Zaragoza, Spain) was performed according to the manufacturer’s instructions. Based on the presence and intensity of the red band, the samples were qualified as negative, positive (intensely colored band), or inconclusive (faint band; Figure 1). Secondly, the samples were examined microscopically following concentration by the flotation technique, as previously described [20]. All samples that were inconclusive or positive by either test were stored in molecular-grade pure ethanol (1 part sample/3 parts ethanol) at −20 °C for further molecular processing.

The DNA was isolated using dedicated commercial kits (ISOLATE II Fecal DNA kit, meridian Bioscience, London, UK), according to the manufacturer’s instructions. The amplification of various target genes of the parasites was performed by nested PCR, using previously published primers and protocols (Table 1). 

Each amplification set included a positive control consisting of pathogen DNA attained and confirmed by sequencing during previous studies [12,24] and one no-template control consisting of PCR-grade water in order to assess possible contamination. The PCR products were visualized by electrophoresis in 2% agarose gels stained with the EcoSafe nucleic acid staining solution (Pacific Image Electronics, New Taipei, Taiwan), and their size was assessed by comparison to a molecular marker (HyperLadder™ 100 bp, meridian Bioscience, UK). All bands of the expected size (Table 1) were excised from the gels, purified using a commercial kit (Gel/PCR DNA Fragments Kit, Geneaid Biotech, New Taipei, Taiwan), and bidirectionally sequenced using an external service (performed by Macrogen Europe B.V., Amsterdam, The Netherlands). The obtained chromatograms were assembled and edited using geneious software (Biomatters Ltd., Auckland, New Zealand), and the consensus sequences were compared to those available in the GenBank^®^ database by means of Basic Local Alignment Search Tool (BLAST) analysis. 

The phylogenetic analyses were conducted using MEGA X (10.2.6) software [25]. The sequences were aligned using the MUSCLE algorithm, and the evolutionary history was inferred by using the Maximum Likelihood method, with models chosen based on the lowest Bayesian Information Criterion (BIC) scores, as follows: Tamura 3-parameter model [26] for *Cryptosporidium* spp.; and Tamura 3-parameter model with a discrete Gamma distribution among sites for *gdh*, Kimura 2-parameter model [27] for *bg*, and Kimura 2-parameter model with a discrete Gamma distribution among sites for *tpi* genes of *Giardia duodenalis*. 

The statistical analyses were performed using EpiInfo™ 7.2 software (CDC, Atlanta, GA, USA). The demographic, clinical, and paraclinical characteristics of the sampled patients were analyzed descriptively. The frequency and prevalence of gastrointestinal parasites were tabulated with 95% Confidence Intervals (CIs). The potential risk factors and associations with non-specific symptoms were evaluated based on answers provided on the questionnaires (Supplementary Materials).

## 3. Results

### 3.1. Study Group

Overall, 426 patients submitted samples for the study: 215 males (50.47%) and 211 females (49.53%). The age ranged between 1 and 93 years, with a mean of 34.49 ± 22.97 years and a median of 35 years. The distribution of age-group categories is detailed in Table 2. The admission diagnosis included digestive tract-related pathology in 91 (21.36%) patients. 

A total of 386 patients (90.61%) answered all items on the questionnaire, 11 (2.58%) provided incomplete answers, and 29 (6.81%) did not fill out any items. According to the available answers, the majority of patients (242; 56.8%) lived in an urban environment, followed by rural (129; 30.28%) and mixed urban/rural (25; 5.86%). The housing was in an apartment for 197 patients (46.24%) and a private house for 199 patients (46.71%), with 79 houses (39.69% of total) not connected to the centralized sewage system.

### 3.2. Initial Screening of Samples

The results of rapid tests are presented in Table 3.

Through a microscopical examination of the samples following the flotation method, a total of 12 samples (2.82%; 95% CI, 1.62–4.86%) were positive, as follows: *Giardia duodenalis* cysts were visualized in 10 samples (2.35%; 95% CI, 1.28–4.27), of which 9 were regarded as positive by antigen testing, and 1 as negative; *Strongyloides* spp. larvae and *Enterobius vermicularis* eggs were also detected in 1 sample each (0.23%; 95% CI, 0.04–1.32).

### 3.3. Molecular Analysis

**Figure 2:**
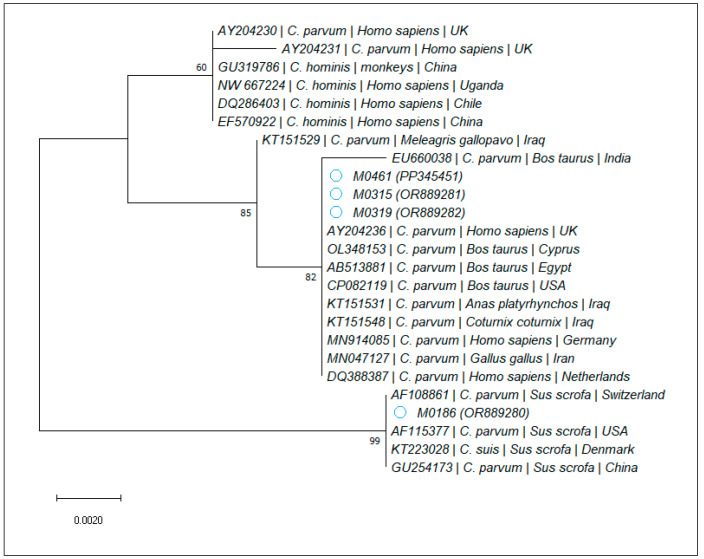
Bootstrap consensus tree obtained from 1000 replicates. The percentage of trees in which the associated taxa clustered together is shown next to the branches. The tree is drawn to scale, with branch lengths measured in the number of substitutions per site. The attributed GenBank Accession Numbers for the sequences obtained during the present study are shown in brackets, next to the sample code.

For *Cryptosporidium* spp., four samples, corresponding to the rapid tests assessed as positive, were confirmed by PCR amplification and sequencing. Through the BLAST analysis, all isolates were identified as *C. parvum*, with one being 100% identical to two isolates obtained from pigs (AF108861, AF115377), while the other three showed 100% nucleotide identity to various *C. parvum* sequences obtained from cattle (e.g., CP141124, CP082119, and OL348153), Broiler chickens (MN047127), and wild ducks (KT151531). The phylogenetic analysis is presented in Figure 2.

**Figure 3:**
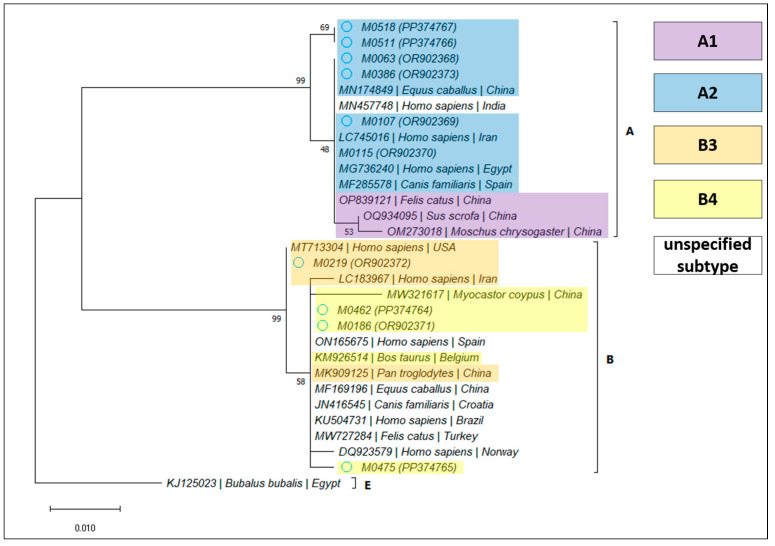
Bootstrap consensus tree obtained from 1000 replicates, based on the *bg* gene of *Giardia duodenalis*. The percentage of trees in which the associated taxa clustered together is shown next to the branches. The tree is drawn to scale, with branch lengths measured in the number of substitutions per site. The attributed GenBank Accession Numbers for the sequences obtained during the present study are shown in brackets, next to the sample code.

**Figure 4:**
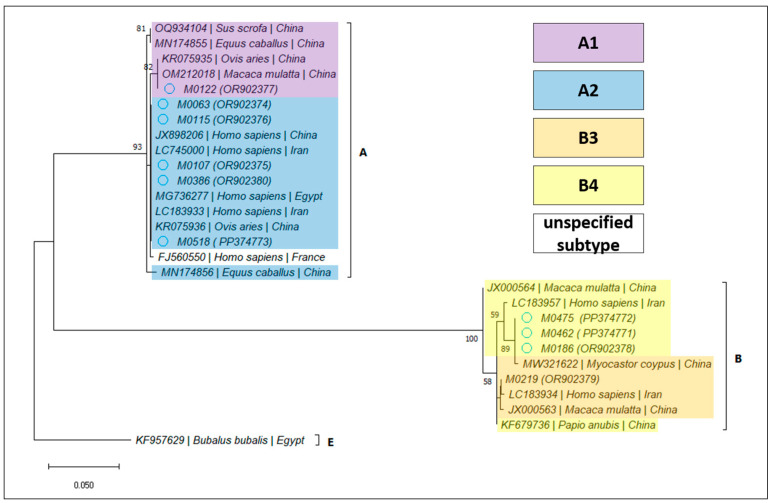
Bootstrap consensus tree obtained from 1000 replicates, based on the *tpi* gene of *Giardia duodenalis*. The percentage of trees in which the associated taxa clustered together is shown next to the branches. The tree is drawn to scale, with branch lengths measured in the number of substitutions per site. The attributed GenBank Accession Numbers for the sequences obtained during the present study are shown in brackets, next to the sample code.

**Figure 5:**
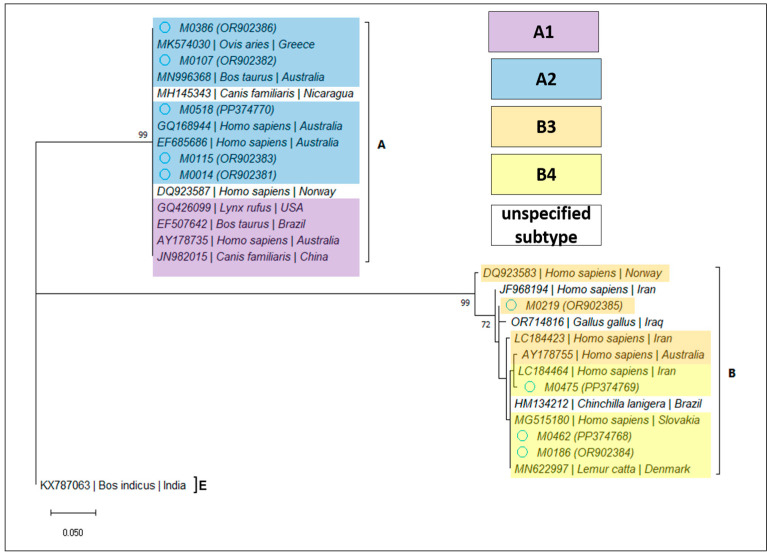
Bootstrap consensus tree obtained from 1000 replicates, based on the *gdh* gene of *Giardia duodenalis*. The percentage of trees in which the associated taxa clustered together is shown next to the branches. The tree is drawn to scale, with branch lengths measured in the number of substitutions per site. The attributed GenBank Accession Numbers for the sequences obtained during the present study are shown in brackets, next to the sample code.

For *Giardia duodenalis*, 12 samples (2.82%; 95% CI 1.62–4.86%) were confirmed by PCR amplification and sequencing, as detailed in Table 4. Based on the BLAST analysis, the determined assemblages and subassemblages are presented in Table 5 and Figure 3, Figure 4 and Figure 5.

### 3.4. Centralization of Results and Statistical Analysis 

Based on the combined results of all employed tests, the overall prevalence of gastrointestinal parasites was of 3.99% (17/426; 95% CI, 2.51–6.3), with *G. duodenalis* accounting for the majority of cases (12/17), followed by *C. parvum* (4/17), while helminths were identified in one patient each. A single coinfection of *G. duodenalis* and *C. parvum* was identified in one immunodepressed patient. 

A great majority of positive patients (12/17) were admitted for digestive tract-related pathology (χ^2^ = 22.58; d.f. = 1; *p* < 0.0001). The asymptomatic patients were immunocompetent adults infected with *G. duodenalis* (Table 6). 

According to the questionnaires, the positive patients reported nausea and vomiting with a significantly higher frequency as compared to negative ones (*p* = 0.0007 and 0.003, respectively). No significant differences were noted for the frequency of other expected symptoms (i.e., bloating, abdominal pain, or diarrhea).

The average CBC results were significantly modified for positive patients in case of total thrombocytes and eosinophils’ ratio (Table 7).

Among all of the investigated potential risk factors, during the present study, only gender was identified as being statistically significant (Table 8).

With regards to clinical evolution, all *Giardia*-confirmed patients were treated with a specific therapy consisting of metronidazole. All four molecularly confirmed *C. parvum* patients were admitted for acute enterocolitis, presenting with numerous diarrheic discharges, abdominal pain, and dehydration. The clinical status of the two immunocompetent patients improved rapidly with supportive therapy. For the immunodepressed patients, specific antimicrobial treatment (nitazoxanide) was recommended.

## 4. Discussion

The present study was conducted as a complex and integrated untargeted parasitological screening of the general population in Northwestern Romania, including all hospitalized patients willing to participate. Due to safety reasons, the only exclusion criterion was COVID-19 infection.

The most prevalent parasite was *G. duodenalis*. Of the total samples, 28 (6.75%) yielded a band indicating positivity for the parasitic antigen. Although the producers’ instructions do not mention the intensity of the test band as interpretation criterion and indicate no cross-reactivity with other microorganisms, we regarded the low-intensity bands as inconclusive (doubtful). By microscopy, no parasitic cysts were visualized in any of the samples classified as inconclusive by rapid testing (19; 4.46%). On the other hand, all of the intensely colored bands (9; 2.11%) were associated with positive microscopy. A single microscopically positive sample yielded a false-negative rapid test result. For the great majority of inconclusive samples (17/19), PCR testing was also negative; therefore, those samples were finally classified as false positives, and the considered true prevalence was 2.82%. 

According to the European Centre for Disease Prevention and Control (ECDC), Romania undertakes passive surveillance of giardiasis, and has reported 5270 cases between 2015 and 2019, but notification rates were not calculated because the national surveillance systems do not cover the whole population [28]. The general prevalence remains mostly unknown in Romania. Between 2004 and 2017, all patients admitted to another tertiary center (n = 54,623) were screened by microscopy regardless of their admission pathology, and the overall prevalence was 4.47%, with annual variations ranging between 0.65 and 16.36% [11]. The study indicated a higher prevalence in urban areas and in the young-adult age group. However, the proportion of asymptomatic patients was not specified, and no molecular analyses were performed. Although with no statistical significance, most likely due to the overall low positivity rate, in the present study, a majority of infections were diagnosed in patients living in rural areas and, according to age group, in young adults.

The genotyping was successful at all three loci in 8/12 samples, at two loci (*bg* and *tpi*) in one sample, and at a single locus (*bg* or *tpi* or *gdh*) in one sample each. The BLAST analysis indicated that assemblage A was more frequent (8/12), among which we identified one case harboring subtype A1, while the rest were A2. The other four samples belonged to assemblage B: subtype B3 in one case, and B4 in the rest. Interestingly, subtype B4 was found exclusively in immunodepressed patients. However, this potential association needs to be further investigated. Our results are in line with previous findings at the regional level [12], but they are divergent from those of other studies performed in Spain and the UK, which indicated that assemblage B was more frequent [29,30,31]. A recent review [5] concluded that assemblage B was the most frequently identified both at the European and world-wide level. In Europe, out of 1658 isolates, 930 belonged to assemblage B, and 714 to assemblage A, of which 466 were classified as subtype A2. The countries in which the dominant assemblage was A were Germany (14/17), Italy (81/152), Poland (2/3), and Portugal (27/32); meanwhile, assemblage B was predominant in Albania (12/22), Belgium (54/72), France (41/50), and Sweden (128/207). Clinically, assemblage B seems to cause a more severe illness in humans [30,32], while assemblage A-infected patients seem more likely to harbor asymptomatic infections; however, multiple factors, including age and immunity, could affect the clinical presentations of *G. duodenalis* genotypes [12,29,30]. In the present study, the severity of clinical manifestations was not evaluated, but similar to other studies, a majority of asymptomatic patients were positive for assemblage A. 

In the case of *Cryptosporidium* infection, only a portion of rapid tests (4/8) were confirmed by molecular analyses. However, this is not necessarily an indication of false-positivity, as low amplification rates of the SSU rRNA were also reported by other authors, who obtained 14 isolates out of 48 stool samples microscopically confirmed by modified Ziehl–Neelsen staining [19]. Therefore, the actual prevalence of this parasite could not be exactly established but was placed between 0.94 and 1.88%. In Romania, screening for *Cryptosporidium* spp. is not included in coproparasitological standard examinations in clinical laboratories; therefore, most studies are surveys based on convenience sampling of hospitalized children or adults, mainly taking into account those suffering from diarrheic syndrome [33]. The officially reported data are unreliable, as shown by the scientific literature published within the same time frame. Recent data obtained from children from the western part of the country indicate a prevalence of infection of 4.26% in 2010, and 7.54% in 2015 [33]. Between 2017 and 2020, in a laboratory from Eastern Romania, 390 samples (3.54%) were positive for gastro-intestinal parasites, with *Cryptosporidium* spp. representing 2.8% of total positives and 0.09% of investigated samples. Furthermore, in 9/11 samples, *Cryptosporidium* spp. oocysts were in association with *G. duodenalis,* but no epidemiological or clinical data were documented [34]. All of the molecularly confirmed isolates were *C. parvum*, in line with data obtained in Western Romania [18,19], and further reinforcing the role of animal hosts in the transmission cycle of this parasite. At the European Union level, in 2021, information regarding *Cryptosporidium* species was available for 934 reported cases, originating from seven countries, of which 96% were caused by *C. parvum* [17]. The clinical status of the immunocompetent patients improved rapidly without specific medication, reinforcing the self-limiting character of the disease [8].

Overall, the present study indicates a low prevalence of food- and water-borne parasitic infections in the general population in Northwestern Romania. Although the collected data suggest an association between gastro-intestinal parasites and rural environments, drinking water from unsanitary sources (springs and wells), and the consumption of unwashed fruits and vegetables, none of these factors was statistically significant, possibly due to the low positivity rate.

## 5. Conclusions

The present study provides new insights into the epidemiological status and clinical implications of gastro-intestinal parasite species and genotypes in Romania that are necessary for an in-depth understanding of the potential zoonotic transmission and improvement of patient care. A low prevalence, generally associated with digestive tract-related symptoms, was identified, while gender was the only confirmed risk factor. Furthermore, our results indicate that a combination of diagnostic tests (i.e., rapid tests, supplemented by microscopical evaluation and molecular tools) would be advisable, as a single method could yield unreliable results.

## Figures and Tables

**Table 1:** Target genes and primers used for PCR.

Parasite	Target Gene	Product (bp)	Primers	Reference
*Giardia duodenalis*	*tpi*	605	AL3543: AAATIATGCCTGCTCGTCG	[21]
			AL3546: CAAACCTTITCCGCAAACC	
		530	AL3544: CCCTTCATCGGIGGTAACTT	
			AL3545: GTGGCCACCACICCCGTGCC	
	*bg*	753	G7: AAGCCCGACGACCTCACCCGCAGTGC	[22]
			G759: GAGGCCGCCCTGGATCTTCGAGACGAC	
		511	B-F: GAACGAACGAGATCGAGGTCCG	
			B-R: CTCGACGAGCTTCGTGTT	
	*gdh*	-	GDHeF: TCAACGTYAAYCGYGGYTTCCGT	
			GDHiR: GTTRTCCTTGCACATCTCC	
		432	GDHiF: CAGTACAACTCYGCTCTCGG	
			GHDiR: GTTRTCCTTGCACATCTCC	
*Cryptosporidium* spp.	*18S* rRNA	-	CrSSU-1: GATTAAGCCATGCATGTCTAA	[23]
			CrSSU-2: TTCCATGCTGGAGTATTCAAG	
		638	CrSSU-3: CAGTTATAGTTTACTTGATAA	
			CrSSU-4: CCTGCTTTAAGCACTCTAATTTTC	

**Table 2:** Age-group distribution of the study group.

Age Group	*n*	%
Child (0–13 years)	111	26.06
Youth (14–24 years)	33	7.75
Young adult (25–44 years)	145	34.04
Middle-aged (45–60 years)	67	15.73
Old adult (61–80 years)	64	15.02
Long-livers (>81 years)	6	1.41
Total	426	100

**Table 3:** The results of rapid tests based on the intensity of the test band.

		*Cryptosporidium*	*Giardia*
Positive	N	4	9
	% (95% CI)	0.94 (0.37–2.39)	2.11 (1.12–3.97)
Inconclusive	N	4	19
	% (95% CI)	0.94 (0.37–2.39)	4.46 (2.87–6.86)
Total	N	8	28
	% (95% CI)	1.88 (0.95–3.66)	6.75 (4.59–9.34)

**Table 4:** Results obtained for *Giardia duodenalis* by the three employed methods.

Antigen	Microscopically Positive	Confirmed by PCR + Sequencing
Positive: 9	9	9
Inconclusive: 19	0	2
Negative: 1	1	1
Total	10	12

**Table 5:** Genotyping of *Giardia duodenalis* isolates: 1 = successful amplification and sequencing; 0 = unsuccessful sequencing or amplification.

Sample	*bg*	*tpi*	*gdh*	Assemblage	Subtype
1	0	0	1	A	A2
2	1	1	0	A	A2
3	1	1	1	A	A2
4	1	1	1	A	A2
5	0	1	0	A	A1
6	1	1	1	B	B3
7	1	1	1	B	B4
8	1	1	1	A	A2
9	1	1	1	B	B4
10	1	1	1	B	B4
11	1	0	0	A	A2
12	1	1	1	A	A2

**Table 6:** The characteristics of *G. duodenalis*- and *C. parvum*-infected patients.

Code	Sex	Age (Years, Category)		GI Pathology	Immune Status	Parasite
M0014	F	69	Old adult	No	Competent	*G. duodenalis* A2
M0063	M	92	Long-livers	Yes	Competent	*G. duodenalis* A2
M0107	M	35	Young adult	No	Competent	*G. duodenalis* A2
M0115	M	26	Young adult	Yes	Competent	*G. duodenalis* A2
M0122	M	29	Young adult	No	Competent	*G. duodenalis* A1
M0186	M	66	Old adult	Yes	Depressed (HIV)	*G. duodenalis* B4
						*C. parvum*
M0219	M	28	Young adult	No	Competent	*G. duodenalis* B3
M0315	F	8	Child	Yes	Competent	*C. parvum*
M0319	F	24	Youth	Yes	Competent	*C. parvum*
M0386	M	35	Young adult	Yes	Competent	*G. duodenalis* A2
M0461	M	75	Old adult	Yes	Depressed (HIV)	*C. parvum*
M0462	M	29	Young adult	Yes	Depressed (HIV)	*G. duodenalis* B4
M0475	M	21	Youth	Yes	Depressed (HIV)	*G. duodenalis* B4
M0511	M	3	Child	Yes	Competent	*G. duodenalis* A2
M0518	M	35	Young adult	No	Depressed (HIV)	*G. duodenalis* A2

**Table 7:** The average CBC results for tested patients, according to parasitic infection status.

	Positive	Negative	F; *p*
RBC (•10^6^)	4.43 ± 0.73	4.37 ± 0.56	0.17; 0.67
Thrombocytes (•10^3^)	216.7 ± 93.27	257.18 ± 89.7	**3.31; 0.06**
Leucocytes (•10^3^)	7.69 ± 4.84	8.65 ± 4.49	0.72; 0.38
%Lymphocytes	24.58 ± 12.81	26.36 ± 14.56	0.24; 0.62
%Monocytes	9.94 ± 4.31	8.44 ± 3.84	2.45; 0.11
%Neutrophils	59.46 ± 19.34	62.64 ± 16.51	0.75; 0.38
%Eosinophils	5.36 ± 9	1.95 ± 3.86	**10.82; 0.001**
%Basophils	0.58 ± 0.57	0.48 ± 0.35	1.05; 0.3

**Table 8:** Prevalence of gastro-intestinal parasites according to categories. * The ‘not specified’ category was not taken into account.

Variable	Category	Positive/Total	% (95% CI)	χ^2^; d.f.; *p* *
Gender	Male	13/215	6.05 (3.26–10.12)	3.7; 1; **0.04**
	Female	4/211	1.9 (0.52–4.78)	
Age group	Child	3/111	2.7 (0.56–7.7)	4.8; 5; 0.44
	Youth	2/33	6.06 (0.74–20.23)	
	Young adult	7/145	4.83 (1.96–9.69)	
	Middle-aged	1/67	1.49 (0.04–8.04)	
	Old adult	3/64	4.69 (0.98–13.09)	
	Long-livers	1/6	16.67 (0.42–64.12)	
Environment	Rural	6/129	4.65 (1.73–9.85)	1.25; 2; 0.53
	Urban	9/242	3.72 (1.71–6.94)	
	Mixed	0/25	0 (0–13.72)	
	Not specified	2/30	6.67 (0.82–22.07)	
Housing	House	8/199	4.02 (1.75–7.77)	0; 1; 1
	Apartment	7/197	3.55 (1.44–7.18)	
	Not specified	2/30	6.67 (0.82–22.07)	
Outdoor activities	No	3/43	6.98 (1.46–19.06)	1.55; 3; 0.66
	Rarely	5/124	4.03 (1.32–9.16)	
	Weekly	2/65	3.08 (0.37–10.68)	
	Daily	5/164	3.05 (1–6.97)	
	Not specified	2/30	6.67 (0.82–22.07)	
Travelling abroad during the past year	Yes	4/146	2.74 (0.75–6.87)	0.19; 1; 0.58
	No	10/241	4.14 (2.01–7.5)	
	Not specified	3/39	7.69 (1.62–20.87)	
Drinking water	Bottled water	5/159	3.14 (1.03–7.19)	0.88; 2; 0.64
	Tap water	6/162	3.7 (1.37–7.89)	
	Others (well/spring)	4/70	5.71 (1.58–13.99)	
	Not specified	2/35	5.71 (0.7–19.16)	
Eating raw or smoked meat and products	Yes	11/318	3.46 (1.94–6.09)	0.22; 1; 0.49
	No	4/73	5.48 (1.51–13.44)	
	Not specified	2/35	5.71 (0.7–19.16)	
Drinking unpasteurized milk	Yes	4/88	4.55 (1.25–11.32)	0.006; 1; 0.75
	No	11/303	3.63 (2.04–6.38)	
	Not specified	2/35	5.71 (0.7–19.16)	
Eating unwashed fruits and vegetables	Never	7/223	3.14 (1.27–6.36)	4.69; 3; 0.19
	Sometimes	8/121	6.61 (2.9–12.61)	
	Frequently	0/40	0 (0–9.81)	
	Almost always	0/7	0 (0–40.96)	
	Not specified	2/35	5.71 (0.7–19.16)	
Washing hands before a meal	Never	0/1	0 (0–97.5)	0.54; 3; 0.9
	Sometimes	1/49	2.04 (0.05–10.85)	
	Frequently	4/102	3.92 (1.08–9.74)	
	Almost always	10/239	4.18 (2.02–7.56)	
	Not specified	2/35	5.71 (0.7–19.16)	
Owning pets and/or farm animals	Yes	9/238	3.78 (1.74–7.06)	0; 1; 1
	No	6/153	3.92 (1.45–8.34)	
	Not specified	2/35	5.71 (0.7–19.16)	

## Data Availability

The relevant data supporting the conclusions of this article are provided within the article and Supplementary Materials. The complete dataset used and analyzed during the current study is available from the corresponding author upon reasonable request.

## Supplementary Materials

The following supporting information can be downloaded at: https://www.mdpi.com/article/10.3390/microorganisms12040762/s1, Applied questionnaire (translated from Romanian).
