# Supplementary material for: Prevalence and Genotyping of Water- and Food-Borne Parasitic Protozoans (Giardia duodenalis and Cryptosporidium spp.) in Hospitalized Patients from Northwestern Romania
Source: Microorganisms. 2024 Apr 10;12(4):762. doi: 10.3390/microorganisms12040762 (PMC11052221; doi:10.3390/microorganisms12040762)
Supplement: Supplementary file 1 [file microorganisms-12-00762-s001.zip › microorganisms-2947616-supplementary.pdf]

QUESTIONNAIRE

Name**______________________________**

Personal Numeric Code ☐☐☐☐☐☐☐☐☐☐☐☐☐ **Phone** _____________________

☐ *I am the participant*

☐ *I am the parent or guardian of the participant*

Occupation ___________________________________

International travelling during the past year?

☐ no ☐ yes where ?______________________________

Persons in the household ____________, of which children ___________

Children in collectivity (kindergarten, school, etc.) ☐ no ☐ yes

Professional or recreational outdoor activities?

☐ daily ☐ weekly ☐ rarely ☐ never

Household ☐ city ☐ village ☐ mixed

☐ apartment ☐ house

If living in a house, the sewage system is ☐ centralized ☐ own, uncentralized

Main source of drinking water:

☐ tap water ☐ bottled ☐ other sources (well/spring)

Consumption of raw milk?

☐ no ☐ yes

Consumption of raw or undercooked (rare) meat?

☐ no ☐ yes

Consumption of raw or smoked meat products (sausages, pastrami, etc.)?

☐ no ☐ yes

Consumption of unwashed fruits and vegetables?

☐ no ☐ sometimes ☐ frequently ☐ almost always

Washing hands before a meal?

☐ no ☐ sometimes ☐ frequently ☐ almost always

Owning the following animals?

	No	Yes	Indoor access
Dog(s)			
Cat(s)			
Hamster(s)			
Fish			
Turtle(s) or other reptiles			
Cage bird(s)			
Rabbit(s)			
Cow(s)			
Horse(s)			
Sheep			
Goat(s)			
Poultry			
Others?			

Have you ever been diagnosed with a parasitic disease?

☐ no ☐ yes, which? ______________________________

Were you prescribed or did you take antiparasitic medication?

☐ no ☐ yes, which product/substance?______________________________

Any recent contact with persons having parasitic diseases?

☐ no ☐ yes ☐ I don’t know

Do you have one or more of the following symptoms for no apparent reason? (check all that apply)

	**No (0)**	**Yes**			
		Sometimes (1) (less than 1/month)	Rarely (2) (1-2/month)	Frequently (3) (1/week)	Very frequently (4) (>1/week)
Bloating					
Loss of appetite					
Nausea					
Vomiting					
Abdominal pain and/or cramps					
Constipation					
Diarrhea					
Weight loss					
Fever					
Fatigue					
Headaches					
Dizziness					
Muscle pain					
Joint pain					
Coughing					
Nettle rash					
Others:					

Signature ____

Date ______________
